# Pressurized water reactor spent nuclear fuel data library produced with the Serpent2 code

**DOI:** 10.1016/j.dib.2020.106429

**Published:** 2020-10-20

**Authors:** Zsolt Elter, Li Pöder Balkeståhl, Erik Branger, Sophie Grape

**Affiliations:** Uppsala University, Sweden

**Keywords:** Spent nuclear fuel, Fuel library, Nuclear safeguards, Serpent, Material composition

## Abstract

The paper describes a data library containing material composition of spent nuclear fuel. The data is extracted from burnup and depletion calculations with the Serpent2 code. The simulations were done with a PWR fuel pin cell geometry, for both initial UO_2_ and MOX fuel load for a wide range of initial enrichments (IE) or initial plutonium content (IPC), discharge burnup (BU) and cooling time (CT).

The fuel library contains the atomic density of 279 nuclides (fission products and actinides), the total spontaneous fission rate, total photon emission rate, activity and decay heat at 789,406 different BU, CT, IE configurations for UO_2_ fuel and at 531,991 different BU, CT, IPC configurations for MOX fuel. The fuel library is organized in a publicly available comma separated value file, thus its further analysis is possible and simple.

## Specifications Table

**Table:** 

Subject	Nuclear Energy and Engineering
Specific subject area	Nuclear spent fuel characterization and nuclear safeguards
Type of data	Table
How data were acquired	Computer simulations with the Serpent 2.1.28 Continuous-energy Monte Carlo Reactor Physics Burnup Calculation Code
Data format	Raw, Filtered
Parameters for data collection	Fuel assembly geometry: PWR pincell (dimensions detailed later) Fuel material type:Uranium oxide (UO_2_) and Mixed Oxide (MOX) Initial enrichment (UO_2_): 1.5% to 6.0% in steps of 0.1% Initial plutonium content (MOX): 4.0% to 10.0% in steps of 0.2% Discharge burnup: 5 MWd/kgHM to 70 MWd/kgHM in steps of 0.5 MWd/kg Cooling time: 0 years to 70 years in steps of 0.25 years between 0 and 10 years, in steps of 0.5 years between 10 and 40 years and in steps of 1 year between 40 and 70 years.
Description of data collection	The data was extracted from output files of Serpent2 simulations.
Data source location	Uppsala, Sweden
Data accessibility	Repository name: Uppsala University Pressurized water reactor spent nuclear fuel data library, Mendeley Data, v1 Data identification number: doi:10.17632/8z3smmw63p.1 Direct URL to data: http://dx.doi.org/10.17632/8z3smmw63p.1
Related research article	S. Grape, E. Branger, Zs. Elter, L. Pöder Balkeståhl, Determination of spent nuclear fuel parameters using modelled signatures from non-destructive assay and Random Forest regression, Nuclear Instruments and Methods in Physics Research Section A, 10.1016/j.nima.2020.163979

## Value of the Data

• The fuel library constitutes an extensive collection of spent fuel inventories covering well-defined and structured operational histories. Due to the large number of included IE, BU and CT values the spent fuel samples are ideal for data analysis with machine learning techniques.
• The fuel library can be used for educational purposes to demonstrate and exemplify the evolution of various isotopes in the fuel over its lifetime. The fuel library can also be used for research purposes where, for instance, spent nuclear fuel assemblies are characterized or assessed based on fuel parameters or content.
• The fuel library enables an evaluation of various detector responses since the amount of detected radiation from the spent fuel is related to its isotopic composition.
• The fuel library complements the efforts of SCK CEN [3], who published a similar fuel library obtained with other depletion codes.

## 1 Data Description

The fuel library contains the simulated nuclide inventory of an irradiated PWR uranium-dioxide (UO_2_) fuel cell for various BUs, CTs and IEs. Similarly, the nuclide inventory has been modelled for mixed oxide (MOX) fuel with the same geometry for various IEs, BUs and initial plutonium contents. The fuel library is stored in a comma-separated values file, named ´UU_PWR_UOX-MOX.csv´. The file has 288 columns and 1,321,397 rows in addition to the header row. 789,406 rows describe UO_2_ inventories (131 BU steps, 131 CT steps and 46 IE steps as described in the Specifications table). 531,991 rows describe MOX inventories (131 BU steps, 131 CT steps and 31 IE steps as described in the Specifications table). The first column contains an index, the next 8 columns are presented in Table 1. Note that the spontaneous fission rate, gamma source rate, activity and decay heat are given on a per axial length basis (in cm units), since the calculations are performed in 2D.

**Table 1:** Description of columns in the fuel library.

Column name	Explanation
‘BU’	Discharge burnup value in MWd/kgU
‘CT’	Cooling time in days
‘IE’	Initial enrichment for UO_2_ and initial plutonium content (%(Pu + Am) / Heavy metal) for MOX
‘fuelType’	Description of the fuel type. Takes the value of ‘UOX’ or ‘MOX’ for UO_2_ and MOX fuel, respectively.
‘TOT_SF’	Spontaneous fission rate in fissions per second on a per axial length basis
‘TOT_GSRC’	Photon emission rate in photons per second on a per axial length basis
‘TOT_A’	Activity in Becquerels on a per axial length basis
‘TOT_H’	Decay heat in Watts on a per axial length basis

The next 279 columns contain atomic densities in [10^24^/cm^3^] units for 279 different nuclides, each named with their respective chemical element and the mass number concatenated. When applicable the metastable state is highlighted with “m” at the end of the string. The included nuclides are the following given by the column names:

'H1′, 'H2′, 'H3′, 'He3′, 'He4′, 'Li6′, 'Li7′, 'Be9′, 'B10′, 'B11′, 'C12′, 'N14′, 'N15′, 'O16′, 'O17′, 'Ga69′, 'Ga71′, 'Ge70′, 'Ge72′, 'Ge73′, 'Ge74′, 'Ge76′, 'As74′, 'As75′, 'Se74′, 'Se76′, 'Se77′, 'Se78′, 'Se79′, 'Se80′, 'Se82′, 'Br79′, 'Br81′, 'Kr78′, 'Kr80′, 'Kr82′, 'Kr83′, 'Kr84′, 'Kr85′, 'Kr86′, 'Rb85′, 'Rb86′, 'Rb87′, 'Sr84′, 'Sr86′, 'Sr87′, 'Sr88′, 'Sr89′, 'Sr90′, 'Y89′, 'Y90′, 'Y91′, 'Zr90′, 'Zr91′, 'Zr92′, 'Zr93′, 'Zr94′, 'Zr95′, 'Zr96′, 'Nb93′, 'Nb94′, 'Nb95′, 'Mo92′, 'Mo94′, 'Mo95′, 'Mo96′, 'Mo97′, 'Mo98′, 'Mo99′, 'Mo100′, 'Tc99′, 'Ru98′, 'Ru99′, 'Ru100′, 'Ru101′, 'Ru102′, 'Ru103′, 'Ru104′, 'Ru105′, 'Ru106′, 'Rh103′, 'Rh105′, 'Pd102′, 'Pd104′, 'Pd105′, 'Pd106′, 'Pd107′, 'Pd108′, 'Pd110′, 'Ag107′, 'Ag109′, 'Ag111′, 'Ag110m', 'Cd106′, 'Cd108′, 'Cd110′, 'Cd111′, 'Cd112′, 'Cd113′, 'Cd114′, 'Cd115′, 'Cd116′, 'Cd115m', 'In113′, 'In115′, 'Sn112′, 'Sn113′, 'Sn114′, 'Sn115′, 'Sn116′, 'Sn117′, 'Sn118′, 'Sn119′, 'Sn120′, 'Sn122′, 'Sn123′, 'Sn124′, 'Sn125′, 'Sn126′, 'Sb121′, 'Sb123′, 'Sb124′, 'Sb125′, 'Sb126′, 'Te120′, 'Te122′, 'Te123′, 'Te124′, 'Te125′, 'Te126′, 'Te127′, 'Te128′, 'Te129′, 'Te130′, 'Te132′, 'Te127m', 'Te129m', 'I127′, 'I129′, 'I130′, 'I131′, 'I135′, 'Xe126′, 'Xe128′, 'Xe129′, 'Xe130′, 'Xe131′, 'Xe132′, 'Xe133′, 'Xe134′, 'Xe135′, 'Xe136′, 'Cs133′, 'Cs134′, 'Cs135′, 'Cs136′, 'Cs137′, 'Ba132′, 'Ba133′, 'Ba134′, 'Ba135′, 'Ba136′, 'Ba137′, 'Ba138′, 'Ba140′, 'La138′, 'La139′, 'La140′, 'Ce138′, 'Ce139′, 'Ce140′, 'Ce141′, 'Ce142′, 'Ce143′, 'Ce144′, 'Pr141′, 'Pr142′, 'Pr143′, 'Nd142′, 'Nd143′, 'Nd144′, 'Nd145′, 'Nd146′, 'Nd147′, 'Nd148′, 'Nd150′, 'Pm147′, 'Pm148′, 'Pm149′, 'Pm151′, 'Pm148m', 'Sm144′, 'Sm147′, 'Sm148′, 'Sm149′, 'Sm150′, 'Sm151′, 'Sm152′, 'Sm153′, 'Sm154′, 'Eu151′, 'Eu152′, 'Eu153′, 'Eu154′, 'Eu155′, 'Eu156′, 'Eu157′, 'Gd152′, 'Gd153′, 'Gd154′, 'Gd155′, 'Gd156′, 'Gd157′, 'Gd158′, 'Gd160′, 'Tb159′, 'Tb160′, 'Dy156′, 'Dy158′, 'Dy160′, 'Dy161′, 'Dy162′, 'Dy163′, 'Dy164′, 'Ho165′, 'Ho166m', 'Er162′, 'Er164′, 'Er166′, 'Er167′, 'Er168′, 'Er170′, 'U232′, 'U233′, 'U234′, 'U235′, 'U236′, 'U237′, 'U238′, 'U239′, 'U240′, 'U241′, 'Np235′, 'Np236′, 'Np237′, 'Np238′, 'Np239′, 'Pu236′, 'Pu237′, 'Pu238′, 'Pu239′, 'Pu240′, 'Pu241′, 'Pu242′, 'Pu243′, 'Pu244′, 'Am241′, 'Am242′, 'Am243′, 'Am244′, 'Am242m', 'Am244m', 'Cm240′, 'Cm241′, 'Cm242′, 'Cm243′, 'Cm244′, 'Cm245′, 'Cm246′, 'Cm247′, 'Cm248′, 'Cm249′, 'Cm250′, 'Cf249′, 'Cf250′, 'Cf251′, 'Cf252′, 'Cf253′, 'Cf254′

It is possible to filter the fuel library further by setting conditions on the burnup, cooling time, initial enrichment and fuel type. Note, that loading the whole fuel library requires a large amount of memory, thus most personal computers will experience difficulties when the library is opened with a software which displays the values (e.g. spreadsheet or text editor applications). Thus, it is advised to load only the columns which are of interest for a given analysis. The below example presents a way to load certain columns of the data and then apply conditions with the pandas library [1] in python:

import pandas as pd

fueldata=pd.read_csv('UU_PWR_UOX-MOX.csv',header = 0,

   usecols=['BU','CT','IE','fuelType','TOT_SF','TOT_GSRC','Cs137′])

fuedata_subset=fueldata[(fueldata['BU']==50.0) & (fueldata['IE']==3.5) & (fueldata['fuelType']=='UOX')]

The details of the Serpent2 input files which were used to create the fuel library are given in Table 2. The plutonium and uranium vector which were used to describe the MOX fuel is given in Table 3.

**Table 2:** Details of the Serpent2 input.

Fuel pellet radius (cm)	0.41
Clad inner radius (cm)	0.42
Clad outer radius (cm)	0.48
Pitch between pins (cm)	1.26
Fuel material	Low enriched UO_2_ or MOX
Fuel density (g/cm^3^)	10.5
Fuel temperature (K)	1500
Cladding material	Natural Zirconium
Cladding density (g/cm^3^)	6.52
Cladding temperature (K)	900
Coolant material	Pressurized water
Coolant density (g/cm^3^)	0.75
Coolant temperature (K)	600
Boundary condition	Reflective (set bc 2)
Number of histories per generation Number of active generations Number of inactive generations	5000 100 10 (set pop 5000 100 10)
Power density (kW/g)	27.397⋅10^−3^ (set powdens 27.39726027e–3)
Operational history	10 MWd/kgHM burnup each year (365 days) calculated in 0.5 MWd/kgHM steps. After every 10 MWd/kgHM burnup period, a 30 days downtime (set powdens 0) is included. Burnup goes up to 70 MWd/kgHM (i.e. 7 years of operation)
Neutron cross section library Decay and fission yield data library	JEFF 3.1 ENDF-B-VI-8

**Table 3:** Plutonium and uranium vector in MOX fuel.

Plutonium vector (w%)		Uranium vector (w%)	
Pu-238	2.5	U-234	0.0012
Pu-239	54.7	U-235	0.25
Pu-240	26.1	U-238	99.7488
Pu-241	9.5		
Pu-242	7.2		

## 2 Experimental Design, Materials and Methods

### 2.1 Materials

The Serpent2 computer code was used to calculate the nuclide inventory of irradiated and cooled nuclear fuel. Serpent2 is a Monte Carlo code, which has burnup calculation capabilities [2]. Serpent2 requires the user to write textual input files describing the geometry and the material properties of the investigated problem.

**Fig. 1:**
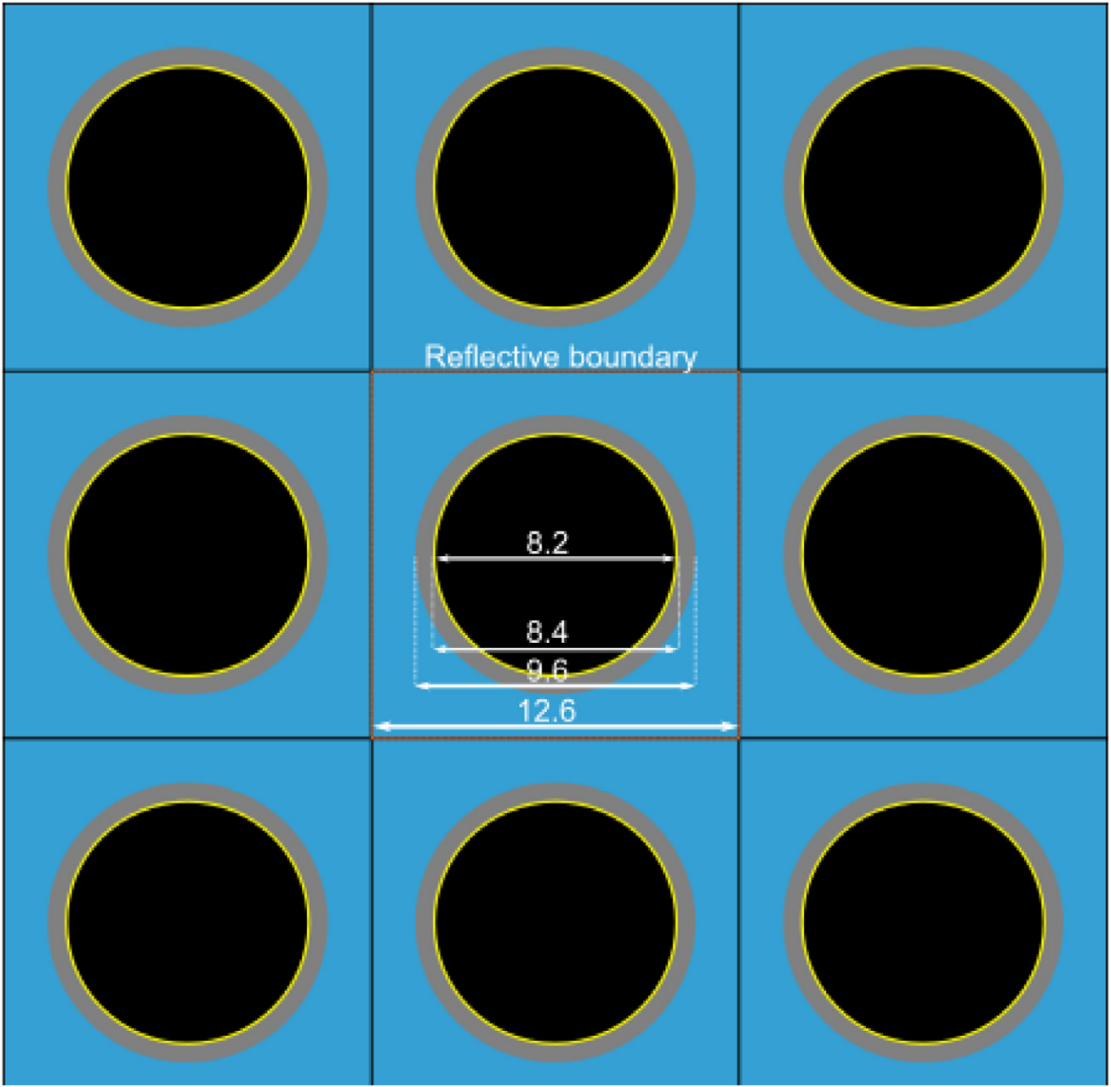
PWR pin cell lattice with the dimensions of fuel, cladding (in/out) and pitch.

In this work an axially infinite 2D PWR pin cell model with reflected boundary conditions was used to create the fuel library. The simulated geometry is shown in Fig. 1 and the details of the model are summarized in Table 2. Note that in case of some parameters such as fuel density the same value was used for both the UO_2_ and MOX case, however in practice such parameters might be slightly different.

In case of MOX fuel, the plutonium and uranium vectors were assumed to be the same as in [3] and is summarized in Table 3.

### 2.2 Method

Traditionally, depletion codes are run for a number of irradiation cycles and cooling times, each of those defined as consisting of several BU steps and CT steps. The depletion code typically outputs the radionuclide inventory after each BU and CT step, which would require one depletion calculation to be performed for each BU-CT combination with all time steps included (here 131 × 131 = 17,161 simulations would be required for both the UO_2_ and MOX cases at a given IE). However, one has to note that performing a depletion calculation up to a specific BU value requires the calculation of any lower BU values. With this approach, a vast amount of depletion calculations are essentially repeated.

Since depletion calculations at different BU steps are time consuming, whereas calculating the CT steps is effortless, we have used a different approach that reduces the computational needs. For each IE value, one depletion calculation is performed up to a discharge BU of 70 MWd/kgU with BU steps of 0.5 MWd/kgU. Then, the radionuclide inventory at different CT values is calculated after each BU step in a separate calculation. Separating the burnup and cooling calculations in such a way requires only 46 and 31 (i.e. the number of IE steps) separate depletion calculations for the UO_2_ and MOX cases, respectively. The process can be summarized with the following algorithm:

for IE in InitialEnrichments:

  create burnup inputfile with IE

  run burnup inputfile with IE

  for BU in BurnUps[BurnUps>=5.0]:

   extract inventory from burnup output with IE at BU

   create cooling input with inventory at BU

   run cooling input with inventory at BU

   for CT in CoolingTimes:

     extract inventory after CT

     append to fuel library

The only disadvantage with this strategy is the fact that the same seed is used for all depletion calculations, thus the radionuclide inventory values are correlated. However, it was investigated that the associated random errors in the radionuclide inventories (and thus also their correlations) are negligible considering the number of neutron histories and cycles included in the simulations.

The radionuclide inventory is calculated at 131 × 131 × 46 = 789,406 grid points corresponding to 131 different BU values, 131 different CT values and 46 different IE values for UO_2_ fuel, and at 131 × 131 × 31 = 531,991 grid points corresponding to 131 different BU values, 131 different CT values and 31 different IPC values for MOX fuel.

## Declaration of Competing Interest

None.
